# A Case Report of Tooth Wear Associated with a Patient's Inappropriate Efforts to Reduce Oral Malodor Caused by Endodontic Lesion

**DOI:** 10.1155/2009/727481

**Published:** 2010-02-07

**Authors:** Masahiro Yoneda, Hatsumi Uchida, Nao Suzuki, Mariko Mine, Tomoyuki Iwamoto, Yosuke Masuo, Toru Naito, Yuko Hatano, Takao Hirofuji

**Affiliations:** ^1^Section of General Dentistry, Department of General Dentistry, Fukuoka Dental College, 2-15-1 Tamura, Sawara-ku, Fukuoka 814-0193, Japan; ^2^Dental Hygienist Division, Fukuoka Dental College Medical and Dental Hospital, 2-15-1 Tamura, Sawara-ku, Fukuoka 814-0193, Japan; ^3^Section of Gerodontology, Department of General Dentistry, Fukuoka Dental College, 2-15-1 Tamura, Sawara-ku, Fukuoka 814-0193, Japan

## Abstract

Here, we report a case of severe tooth wear associated with a patient's inappropriate efforts to reduce oral malodor. A 72-year-old male patient visited our breath clinic complaining of strong breath odor. Former dentists had performed periodontal treatments including scaling and root planing, but his oral malodor did not decrease. His own subsequent breath odor-reducing efforts included daily use of lemons and vinegar to reduce or mask the odor, eating and chewing hard foods to clean his teeth, and extensive tooth brushing with a hard-bristled toothbrush. Oral malodor was detected in our breath clinic by several tests, including an organoleptic test, portable sulphide monitor, and gas chromatography. Although patient's oral hygiene and periodontal condition were not poor on presentation, his teeth showed heavy wear and hypersensitiving with an unfitted restoration on tooth 16. Radiographic examination of the tooth did not reveal endodontic lesion, but when the metal crown was removed, severe pus discharge and strong malodor were observed. When this was treated, his breath odor was improved. After dental treatment and oral hygiene instruction, no further tooth wear was observed; he was not concerned about breath odor thereafter.

## 1. Introduction

Patients often worry about the odor of their breath [1]. Oral malodor is primarily associated with the condition of the oral cavity, including the oral hygiene level and periodontal condition [2–4] and is mainly the result of the microbial metabolism of amino acids in local debris [5]. Many of the major compounds that contribute to oral malodor are volatile sulfur compounds (VSCs) such as hydrogen sulfide and methyl mercaptan [6, 7]. Additionally, methylamine, dimethylamine, propionic acid, butyric acid, indole, scatole, and cadaverine have been reported to cause oral malodor [8, 9]. To evaluate the level of oral malodor in patients complaining of halitosis, VSC levels have typically been measured, along with an organoleptic test [10]. 

To diagnose halitosis, a simple classification with corresponding treatment needs has been developed [11, 12], which includes the categories of genuine halitosis, pseudo-halitosis, and halitophobia. Genuine halitosis is subclassified as physiological or pathological halitosis, and pathological halitosis is subclassified as oral or nonoral pathological halitosis. Oral pathological halitosis is caused largely by periodontal disease [13], and its treatment requires periodontal treatment in addition to dental and oral care, oral hygiene instruction, and counseling. Additionally, dental treatment may be necessary to correct faulty restorations that could contribute to poor oral health [12]. 

In this case report, we describe a case of oral pathological halitosis caused by an endodontic lesion, which was overlooked by some dentists. Consequently, the patient's oral malodor did not decrease even after dentist visits. The patient then started his own breath odor-reducing methods, which not only were ineffective but also caused extensive tooth wear.

## 2. Case Report

A 72-year-old male came to our hospital complaining of oral malodor. He first noticed his oral malodor because a family member had pointed out his bad breath about 3 years previously. He visited some dental clinics to address this problem and received some periodontal treatment, including scaling and root planing. However, his oral malodor did not decrease. He attempted using lemons and vinegar to reduce or mask his breath odor, drinking cola to freshen his breath, chewing hard foods (such as plum seeds) for long periods to clean his mouth, and brushing his teeth with a large, hard-bristled brush. 

When the patient came to our breath clinic, we first measured his breath odor as previously described [14, 15]. The severity of oral malodor in this patient was determined using an organoleptic test [10], portable sulphide monitoring (MS-Halimeter E; Interscan Corporation, Chatsworth, CA, USA) [3], and gas chromatography (model GC14B; Shimadzu Works, Kyoto, Japan) as described before [16]. The organoleptic score (OLS) was 4 (tolerable bad smell) according to the classification [11, 12], and all the VSCs such as hydrogen sulphide, methyl mercaptan, and dimethyl sulphide were above positive levels (data not shown).

**Figure 1:**
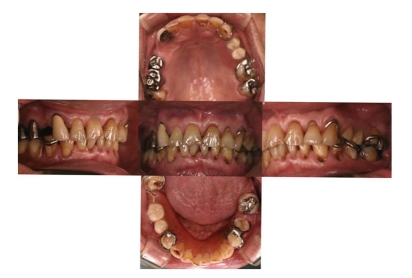
Oral view at the first visit.

**Figure 2:**
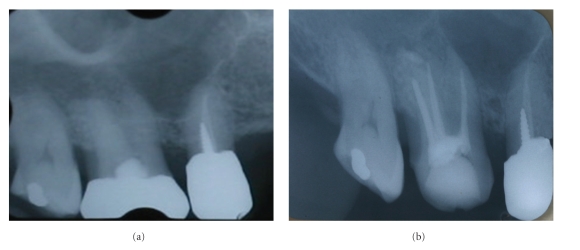
Radiography of tooth 16: (a) before treatment and (b) after root canal filling.

We performed an oral examination to identify the cause of the oral malodor. The oral view at the first visit is shown in Figure 1. Overall periodontal conditions were not poor, except for some periodontal pockets in teeth 26 and 36. These two teeth had 5 mm pockets in the proximal areas (data not shown). Extensive tooth wear was seen in many teeth both on occlusal and cervical surfaces (Figure 1). The tooth surfaces were severely worn and the patient had hypersensitivity in some teeth. There was an unfitted restoration in tooth 16 and we sensed some malodor when compressed air was applied to this tooth. An X-ray of the tooth did not exhibit any periapical lesion, but the root canals seemed poorly treated (Figure 2(a)); so we removed the metal crown and started endodontic treatment. Large amounts of pus accompanied by malodor came from the root canals, but the root canals had improved at the second visit and were filled at the third visit (Figure 2(b)). At this stage, OLS decreased to 2 and we proceeded to other treatment.

**Figure 3:**
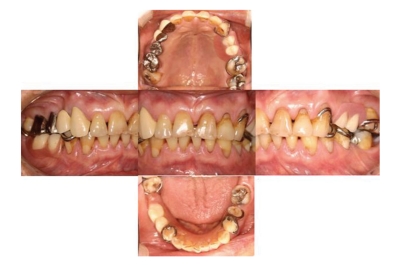
Oral view after treatment.

We explained to the patient the possible causes of oral malodor, such as the unfitted restoration and severe endodontic lesion. We also explained that his tooth wear seemed to be caused by his inappropriate odor-reducing methods such as excessive acid intake and too much brushing. Moreover, he was found to be clenching or grinding his teeth, which may have worsened his tooth wear. We showed him how to brush his teeth without damaging the tooth surfaces and recommended a special toothpaste that prevents tooth wear and aids remineralization of tooth surfaces. We advised him not to clench or grind his teeth and made a night guard to prevent bruxism at night. We then filled or coated the already lost enamel of the teeth (Figure 3).

**Figure 4:**
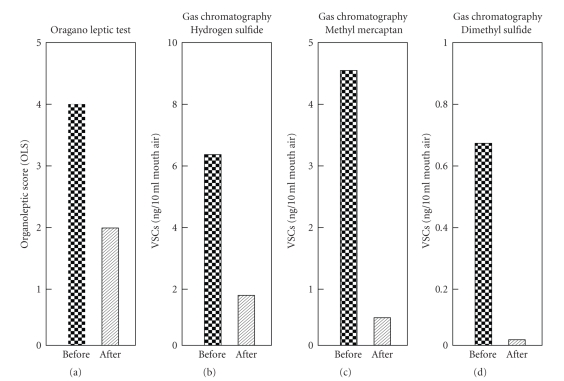
Comparison of breath odor measurements before and after treatment.

Several months later, the patient's breath odor was reduced to the physiological level (Figure 4), and neither he nor his family members had any complaints thereafter about his oral malodor. No new tooth wear occurred during this maintenance period.

## 3. Discussion

The patient in this report had noticed the malodor and visited some dentists, who performed periodontal treatments. While theoe may have been partially effective, the patient's breath odor did not change perceptibly. There was no pain and no periapical lesions, so the patient's endodontic problem may not have been readily apparent. Fortunately, the main cause of his oral malodor was finally identified and treated. After the root canal filling, the OLS decreased to 2, which confirmed that endodontic problem was one of the main causes of his oral malodor. We did not measure with gas chromatography at this stage, because it takes much time to perform the complete breath odor measurement and the organoleptic test is considered reliable and important [11, 12].

There are many causes for oral malodor. Tongue coating, periodontitis, deep caries, and unfitted restorations are main causes of bad breath odor [12]. However, we sometimes encounter other causes, such as internal resorption-associated oral malodor [17], so it is necessary for us to carefully examine and find the actual cause of the malodor.

The number of patients with tooth wear or erosion is increasing [18]. There are several causes of tooth wear; acidic food intake and too much brushing are some of the most common causes. Low-pH foods, including cola or other soft drinks, are known to induce tooth surface loss [18]. Tooth wear used to be considered a physiological aging condition and was left untreated. But today, it is considered a kind of tooth-surface loss syndrome, and composite resin filling or resin coating are strongly recommended [19].

The case reported here indicates the importance of careful examination to find the causes of oral malodor, which may prevent patients from attempting misguided methods of reducing oral malodor.
